# An audit of ranulae occurring with the human immunodeficiency virus infecton

**DOI:** 10.4103/0973-029X.64312

**Published:** 2010

**Authors:** FM Butt, ML Chindia, T Kenyanya, LW Gathece, F Rana

**Affiliations:** *Departments of Oral & Maxillofacial Surgery, University of Nairobi, Kenya*; 1 *Department of Dentistry, Kenyatta National Hospital, Kenya*; 2 *Department of Periodontology, Community and Preventive Dentistry, Kenya*; 3 *Department of Pathology, Aga Khan University Hospital, Nairobi, Kenya*

**Keywords:** Human immunodeficiency virus infection, ranula, periductal lymphocytosis

## Abstract

Currently, published literature has increasingly projected the ranula as a lesion that may be closely associated with exposure to the human immunodeficiency virus (HIV). In this report, we document 28 patients who presented with ranulae, among whom 19 were HIV infected. In some, this was the only lesion that was the sentinel sign of HIV infection. Most probably, this lesion could be considered as one of the clinical markers of this infection.

## INTRODUCTION

Over the years, it has remained most desirable to identify diseases and conditions that could constitute reliable markers of infection with the HIV. Thus diverse investigations worldwide have been done to decipher the sentinel groups of oral mucosal and cutaneo us lesions that could be the defining clinical markers of the emergence and progression of HIV infection.[1] Throughout history of this devastating disease, at least seven cardinal lesions, including oral candidiasis, hairy leukoplakia, Kaposi’s sarcoma, linear gingival erythema, necrotizing ulcerative gingivitis, necrotizing ulcerative periodontitis and non-Hodgkin’s lymphoma, have been internationally identified and characterized as being strongly associated with HIV infection.[2]

Recently, an apparently increased occurrence of ranulae have been documented to have been closely associated with HIV infection.[3] The occurrence of bilateral parotid gland enlargement arising from nonspecific cystic degeneration are lesions that have been consistently associated with exposure to HIV. Indeed, in Eastern Africa, parotid gland enlargement remains an important clinical indicator of the possibility of HIV infection. So far, the pathogenesis of cystic lesions associated with salivary glands in general remains elusive. In this article, we contribute more evidence of ranulae as a possible sentinel sign of HIV infection.

## MATERIALS AND METHODS

This was a prospective study (October 2005 to March 2008) that included all patients who presented with ranulae. The study participants were counseled and consented as per the standard hospital protocol (the Kenyatta National and University of Nairobi Dental Hospitals) prior to inclusion into the study and investigation of their HIV status. During the study period, 28 patients were subjected to standard basic investigations before undergoing surgical treatment and the institution of antiretroviral therapy to those who were HIV infected.

### Findings

**Figure 1:**
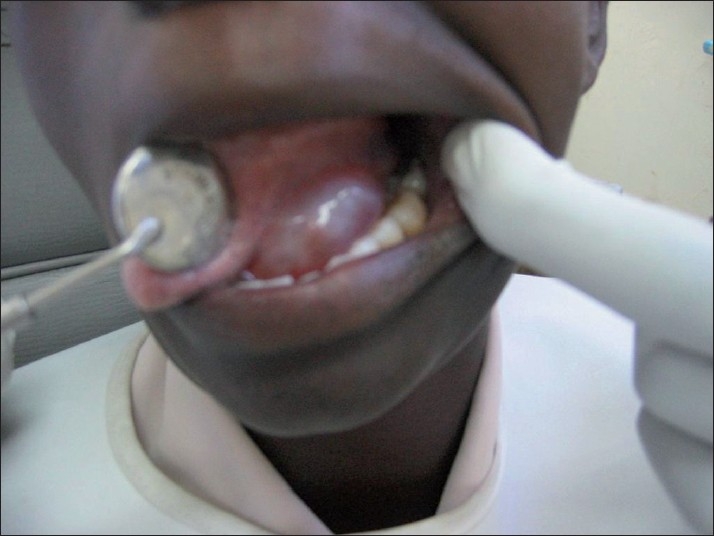
Clinical presentation of a ranula in an HIV-infected patient

**Figure 2:**
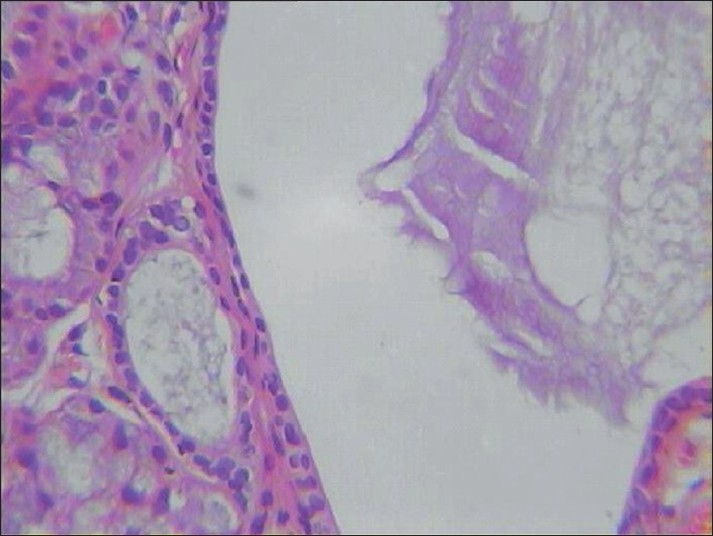
Photomicrograph depicting the histopathological features of ranula (H and E, ×40)

There were a total of 28 patients who presented with ranulae, among whom 19 (67.9%) were HIV-positive and 9 (32.1%) HIV-negative. Their ages ranged between 1.5 and 37 years with a mean of 20.7 years [Table 1]. Among those who were HIV infected, twice as many females presented with ranulae than men, the reverse was true for the non-HIV-infected group. The duration of symptoms varied from two weeks to 36 months. Interestingly, the male cases tended to seek medical attention much earlier than the females. Among the reasons that necessitated patients to seek treatment included difficulty in speech, mastication and deglutition. Lesions which were either unilateral or bilateral varied in size from 2 to 5 cm in their widest of dimensions. The typical clinical appearance of the ranula is illustrated in [Figure 1]. All lesions were surgically extirpated under general anesthesia and subjected to histopathology [Figure 2]. The post-operative follow-up periods varied from 6 to 24 months.

**Table 1:** Age, gender and the HIV-infection status of study participants who presented with ranulae

HIV	Number	Age range (Years)	Average age
Hiv positive			
Males	6	25–37	31.5
Females	13	1.525–28	20.0
Total	19	1.525–37	23.6
Hiv negative			
Males	6	825–37	16.8
Females	3	525–16	9.6
Total		525–37	14.4

## DISCUSSION

Among the various types of oral manifestation associated with HIV-infection and AIDS, there have been some unusual lesions documented in developing regions.[4] Examples of these include Kaposi’s sarcoma, uncommon in some African studies, cheilitis glandularis in Kenya, cancrum oris and ranulae in Zimbabwe and *Penicilliosis marneffei* in South East Asia.[3,5–8] Remarkably, salivary gland diseases (SGD) including salivary gland enlargement, lymphoepithelial cysts and diffuse interstitial lymphocytosis remain important as diagnostic and prognostic indicators of HIV infection.[9] Initially, the sublingual and submandibular salivary glands were often affected; with disease progression, the parotids too got involved.[10] In 1999, Chokunonga reported an increase in the number of cases of ranulae.[11]

In the present study, it is interesting that some of the patients presented with ranula as the initial and the only clinical manifestation of HIV infection. It is difficult to ascertain why there were more females presenting with ranulae than males in the HIV-infected group. Histopathology showed chronic periductal inflammation of the minor salivary gland tissue in all cases. Hyperplasia of goblet cells in mucus glands was also noted. It is possible that this persistent chronic inflammation may lead to small duct obstruction and distension with mucus and subsequent rupture and extravasation of mucus into the surrounding tissues. This extravasation of mucus and loss of lining epithelium was seen in many of our cases. There was no difference noted in histopathological findings among the specimens of the HIV-infected and the non-HIV infected group.

The extravasated mucus may not be cleared by the mononuclear cells in the HIV-infected/AIDS patient as it is well known that these cells are also infected by the virus and there is altered macrophage monocyte functions that entail decreased chemotaxis and phagocytosis. There were no intranuclear inclusions morphologically consistent with cytomegalovirus infection. However, the presence of viruses by other means was not done due to the limitation of resources.

Further case-control studies should be done in future to note the degree of periductal lymphocytosis, isolation of viral particles and chemical analysis of the mucus components of ranulae.

As has been shown by other investigators, the occurrence of ranulae may be considered as a clinical basis upon which the investigation of possible HIV infection may be performed in future. Information on the prevalence of ranulae in the general populations worldwide appears to remain scanty. Therefore, the exact impact of HIV infection on the increased occurrence of this condition may be difficult to determine currently. It is, therefore, important that consistent and comprehensive series of ranulae are presented so that an association of this lesion with HIV infection may be established.
